# Epidemiological measures for assessing the dynamics of the SARS-CoV-2-outbreak: Simulation study about bias by incomplete case-detection

**DOI:** 10.1371/journal.pone.0276311

**Published:** 2022-10-26

**Authors:** Ralph Brinks, Helmut Küchenhoff, Jörg Timm, Tobias Kurth, Annika Hoyer

**Affiliations:** 1 Chair for Medical Biometry and Epidemiology, Faculty of Health/School of Medicine, Witten/Herdecke University, Witten, Germany; 2 Department of Statistics, Ludwig-Maximilians-University Munich, Munich, Germany; 3 Medical Faculty, Institute of Virology, University Hospital Düsseldorf, Düsseldorf, Germany; 4 Institute of Public Health, Charité—Universitätsmedizin Berlin, Berlin, Germany; 5 Biostatistics and Medical Biometry, Medical School OWL, Bielefeld University, Bielefeld, Germany; Universiti Brunei Darussalam Pengiran Anak Puteri Rashidah Sa’adatul Bolkiah Institute of Health Sciences, BRUNEI DARUSSALAM

## Abstract

During the SARS-CoV-2 outbreak, several epidemiological measures, such as cumulative case-counts (CCC), incidence rates, effective reproduction numbers (R_eff_) and doubling times, have been used to inform the general public and to justify interventions such as lockdown. It has been very likely that not all infectious people have been identified during the course of the epidemic, which lead to incomplete case-detection. We compare CCC, incidence rates, R_eff_ and doubling times in the presence of incomplete case-detection. For this, an infection-age-structured SIR model is used to simulate a SARS-CoV-2 outbreak followed by a lockdown in a hypothetical population. Different scenarios about temporal variations in case-detection are applied to the four measures during outbreak and lockdown. The biases resulting from incomplete case-detection on the four measures are compared in terms of relative errors. CCC is most prone to bias by incomplete case-detection in all of our settings. R_eff_ is the least biased measure. The possibly biased CCC may lead to erroneous conclusions in cross-country comparisons. With a view to future reporting about this or other epidemics, we recommend including and placing an emphasis on R_eff_ in those epidemiological measures used for informing the general public and policy makers.

## Introduction

During the global SARS-CoV-2 outbreak starting in late 2019, several epidemiological measures have been used to inform the general public and policy makers. Occasionally, politicians used epidemiological measures to justify interventions such as the obligation to wear face masks or even lockdown. In Germany, for instance, thresholds regarding incidence rates have been implemented into national laws as necessary conditions for relaxation of lockdown, e.g., school-openings or release of dusk-to-dawn curfews [1].

The most prominent epidemiological measure is the cumulative number of people tested positive, which we will call cumulative case-count (CCC). The CCC has been frequently reported on a daily basis on country level (e.g., by the COVID-19 Dashboard of the Johns Hopkins University [2]). To allow cross-country comparisons, CCC or the numbers of people tested positive often have been adjusted for population size, e.g. in the COVID-19 situation reports of the WHO [3]. Some media reported doubling times and effective reproduction numbers (R_eff_). For instance, in a nationally widely perceived press conference Germany’s Chancellor Dr. Angela Merkel declared the political goal to reach doubling greater than ten days [4].

Whether someone is reported as an incident case, is based on the results of the diagnostic procedures carried out. Apart from anamnesis, frequently used diagnostic tests are nasopharyngeal swab tests followed by reverse transcriptase polymerase chain reaction (RT-PCR) to detect viral RNA. If the swab is done properly, RT-PCR has a moderate to high diagnostic sensitivity and a very high specificity for SARS-CoV-2 detection [5]. At least in theory, all incident cases can be identified correctly if enough screening and diagnostic efforts are spent. In practice, of course, only limited resources are available and it is likely that incident cases have been missed. Restrictions in applying diagnostic procedures are, e.g., limitations in the number of available test kits and personnel or the local policy of running diagnostic tests. Local and even national eligibility criteria for tests have been changed during the time of the epidemic. Together with the fact that several people with the associated COVID disease show only asymptomatic or mild courses and presumably have not been considered for diagnosis of an infection at any time, the varying testing frequency leads to the hypothesis that the actual disease was at least partially underestimated [6]. We call an infectious subject who in principle qualifies as a case but has not been detected, because she or he has not been (properly) diagnosed, an undetected case.

A useful measure for assessing case-detection is the proportion of all incident cases that were identified, i.e. the number of detected cases divided by the number of all cases (detected plus undetected cases). This proportion is referred to as the *case detection ratio* (CDR) [7].

The aim of this analysis is to determine the bias of the epidemiological measures used to inform the public and the decision makers due to incomplete case-detection. We conducted a simulation study using the epidemiological parameters of SARS-CoV-2 and mimic incomplete case-detection by assuming scenarios about the CDR. Next, the epidemiological measures are estimated in the presence of incomplete case-detection, and subsequently, the estimations are compared to the true values underlying the simulation.

## Materials and methods

**Fig 1:**
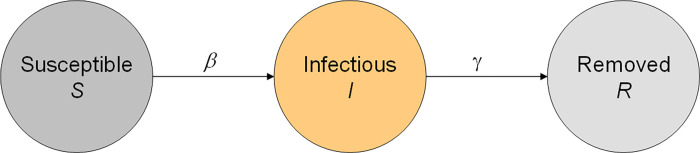
SIR model in infectious disease epidemiology. The population under consideration is divided into three states *susceptible* (prone to be infected), *infected* and *removed* (recovered or dead).

We use the infection-age-structured SIR model [8] to simulate the spread of SARS-CoV-2 in a hypothetical population. In the infection-age-structured SIR as well as in the conventional SIR model, the population is partitioned into three states, the *susceptible* state, the *infected* and the *removed* state. The initial letters of the three states give the model’s name `SIR´ (Fig 1). The infection-age-structured SIR model is a generalization of the SEIR model, which in turn is a generalization of the frequently used SIR model. Compared to the simple SIR, the SEIR comprises an additional latency state (termed ’E’ for *exposed*) when individuals are infected but cannot yet infect others. In this sense, the infection-age-structured SIR model is the most general model which contains SEIR as well as SIR as special cases [8]. We have chosen the infection-aged structured SIR to allow flexibility in the temporal dynamics of transmissibility (for details we refer to the S1 File).

The *removed* state comprises people recovered and deceased from the infected state [9]. As usual in infectious disease epidemiology, changes in numbers of people in the disease states are modeled quantitatively by differential equations, which are presented in the S1 File and can be omitted in favor of keeping the focus on the epidemiological measures used to describe the epidemic. The key part of the model is the transmission rate *β*, which describes the transmission of the virus from an infectious to a susceptible individual at a given time (see the left arrow in Fig 1).

The infection-age-structured SIR model is a minor modification of the SIR model, where the transmission rate *β* depends on two time-scales: the infection age *τ*, i.e., the time since virus transmission from an infectious to a susceptible individual, and calendar time *t*. The latter is often called period in epidemiological contexts. The dependency on the infection age *τ* reflects the evidence that the risk of SARS-CoV-2 transmission depends on the time since infection. Parameters of the infection-age-structured SIR model are chosen according to the best knowledge of the SARS-CoV-2 virus. For introductory texts about the SIR model, we refer the reader to [8, 9] and the references in the S1 File.

Governments of many countries decided to invoke one or more lockdowns, which led us to the idea of simulating three consecutive periods of the epidemic: a phase of increasing number of infections from *t* = 0 to *t* = 25 (days), a phase of implementation of a (strict) lockdown (from *t* = 25 to *t* = 30) followed by a phase post-lockdown when the pandemic is controlled (from *t* = 30 to *t* = 60). Start of implementation of lockdown happens at *t* = 25 (days). We do not make assumptions which specific public health interventions lead to the reduction in transmission e.g. contact tracing, mask wearing, stay-at-home orders, quarantine policies or a combination of these. In this work, we intend to have a realistic dynamic range in the disease transmission rate *β* imposed by public health interventions, irrespective of how these are obtained in detail.

**Fig 2:**
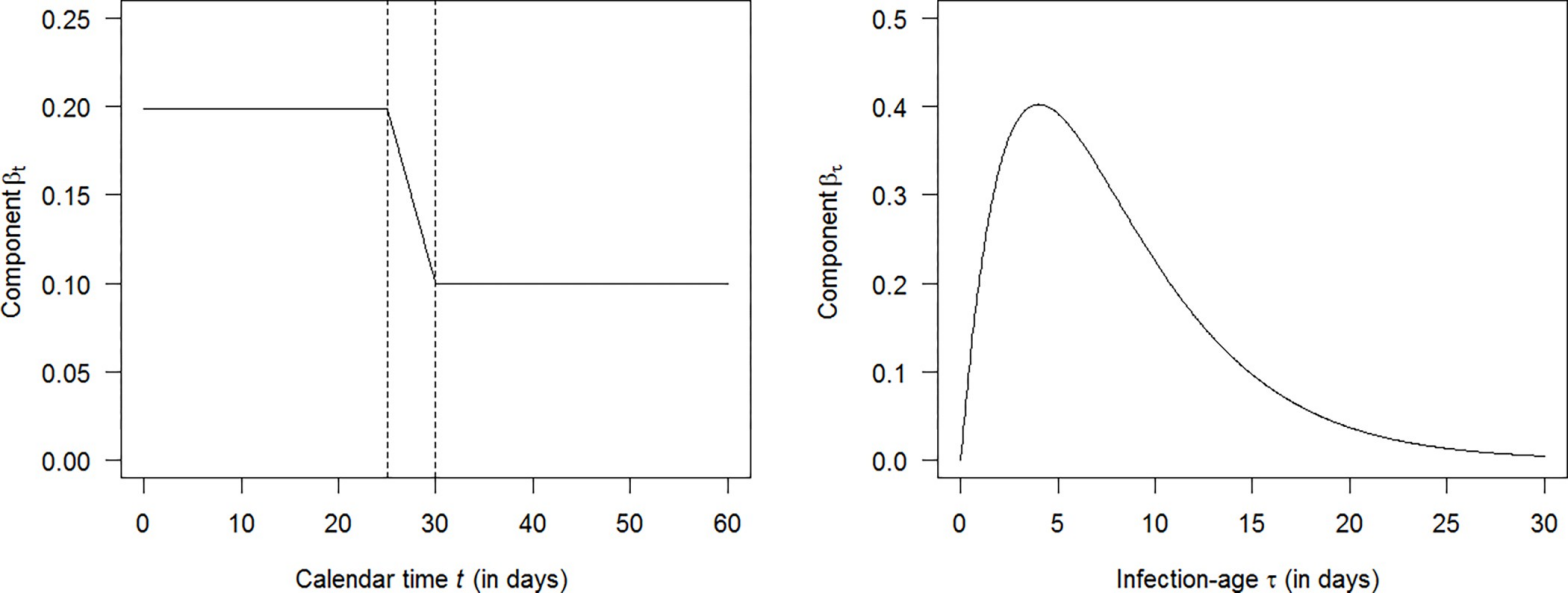
Components of the transmission rate *β*. The left panel shows the time dependent part *β*_**t**_ of the transmission rate *β* during three phases of the pandemic: before lockdown at *t* = 25 (days), installation of lockdown (from *t* = 25 to *t* = 30, between vertical dotted lines) and control of the disease (*t* > 30). The right panel shows the component *β*_**τ**_ of the transmission rate *β* as a function of the infection age *τ*. More details can be found in Section 4 of the **S1** File.

The five day period following the start of the lockdown was chosen as wash-in phase. In real populations, public health interventions usually require some time before taking full effect. After this wash-in period, we assumed that the effect of the lockdown remains unaltered until the end of the simulation. To assess if our findings are robust with respect to the length of the wash-in period and the assumption that effect of the lockdown remains unaltered, we performed sensitivity analyses where we released these assumptions. The results of these sensitivity analyses are presented in Part 1 of the S2 File. Fig 2 shows the marginal distributions of the transmission rate *β*. The left and right part present the dependency on the calendar time *t* and the infection-age τ. Note the steep drop in the transmission rate *β* in the left part of Fig 2 resulting from the lockdown.

All technical details about the model are given in the S1 File. The source code for use in the free Statistical Software R, version 3.6.3 (The R Foundation for Statistical Computing) is available in the Zenodo open public repository [10].

In the case of the novel coronavirus SARS-CoV-2, new infectious cases are often reported daily [2]. If the number of newly incident cases reported on day *t* is denoted by *F*_t_^(o)^ (the superscript ’o’ indicates observed), the case-detection ratio (CDR) is the proportion of these reported cases in relation to the actual (true) but unknown number of newly incident cases *F*_t_ [7]:

$$CDR_{t}=\frac{F_{t}^{\left(o\right)}}{F_{t}}.$$ (1)

If we neglect false positive findings (which is reasonable in highly specific tests), the CDR is a proportion ranging from 0 to 100%. In the case of complete detection of all incident infectious cases on day *t*, the *CDR*_t_ would equal 100% on that day.

We use different scenarios about the temporal variations of the CDR and analyze the biases imposed to the epidemiological measures 1) CCC, 2) incidence rate, 3) R_eff_ and 4) doubling time.

1. The frequently used CCC reported on day *t* simply adds the number of incident cases until day *t*:
$$CCC_{t}=\sum_{s=1}^{t}F_{s}$$ (2)
  Instead of the sum of true (but unknown) incident cases, the observed *CCC*_t_^(o)^ only includes the observed number of incident cases *F*_t_^(o)^.
2. If the daily incidence rates are reported, *F*_t_ and *F*_t_^(o)^ are usually referred to the overall size of the population of the corresponding region, e.g. in the WHO’s weekly epidemiological updates on COVID-19 [3]. In this manuscript, we refer to the incidence rate "per person per day".
3. The effective reproduction number *R*_eff_ is the average number of secondary infectious cases that one primary case infects during their infectious period [11]. *R*_eff_ can be estimated from the number of incident cases *F*_t_. A variety of definitions of the effective reproduction number exist and we refer to the *instantaneous* reproduction number [12].
4. "Doubling times" have often been reported during the temporal course of the pandemic. Originally, the doubling time at some point in time (*t*) is defined as the time span Δ = Δ(*t*) until the number of infectious cases in the population doubles [9]. As the number of infections is not easily accessible, sometimes the "doubling time" refers to the time the incidence [13] or "cumulative incidence doubles" [14]. Thus, we use the defining condition *CCC*_t+Δ_. = 2 *CCC*_t_.

**Fig 3:**
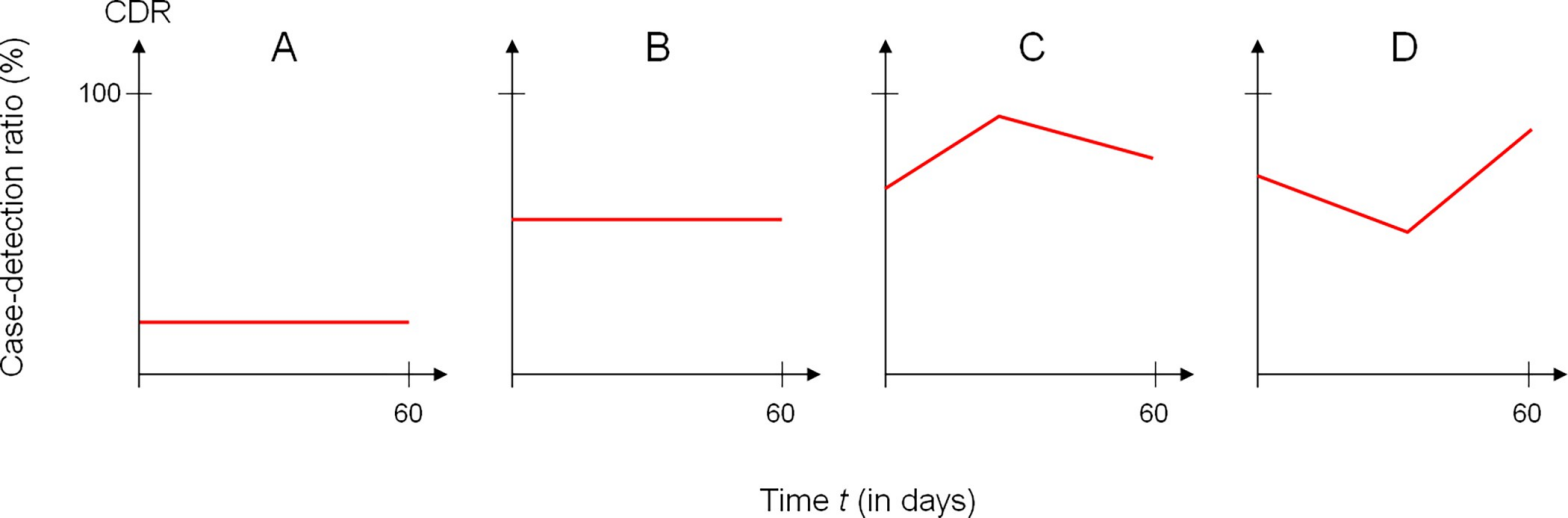
Simulated case-detection ratios (CDRs). CDRs during the pandemic in the four simulated scenarios A to D.

The hypothetical scenarios for the CDR during the pandemic are shown in Fig 3.

In scenarios A and B we assume a low and moderate CDR without temporal trends. These scenarios have been chosen to see if the absolute level of the CDR makes a difference in the four outcome measures. We have not added a scenario with (close to) perfect case detection, because in this case all four epidemiological indices will perform well. In addition, especially in the early days of the pandemic when testing resources have been sparse, a scenario with close high case detection seems unrealistic. Scenarios C and D simulate a CDR that changes over the period of 60 days including a trend reversal at some time. Scenario C mimics the situation when testing capacities are limited at the early phase of a pandemic and are increased after a while, for instance, by investing in resources for testing programs. Scenario D can partly be seen as the opposite of scenario C. After a phase of high CDR in scenario D, testing capacities are increasingly limited for instance by shortage of test supplies. The phase of decreasing CDR is followed by a phase of rising CDR, e.g. by having overcome shortage of test supplies.

**Fig 4:**
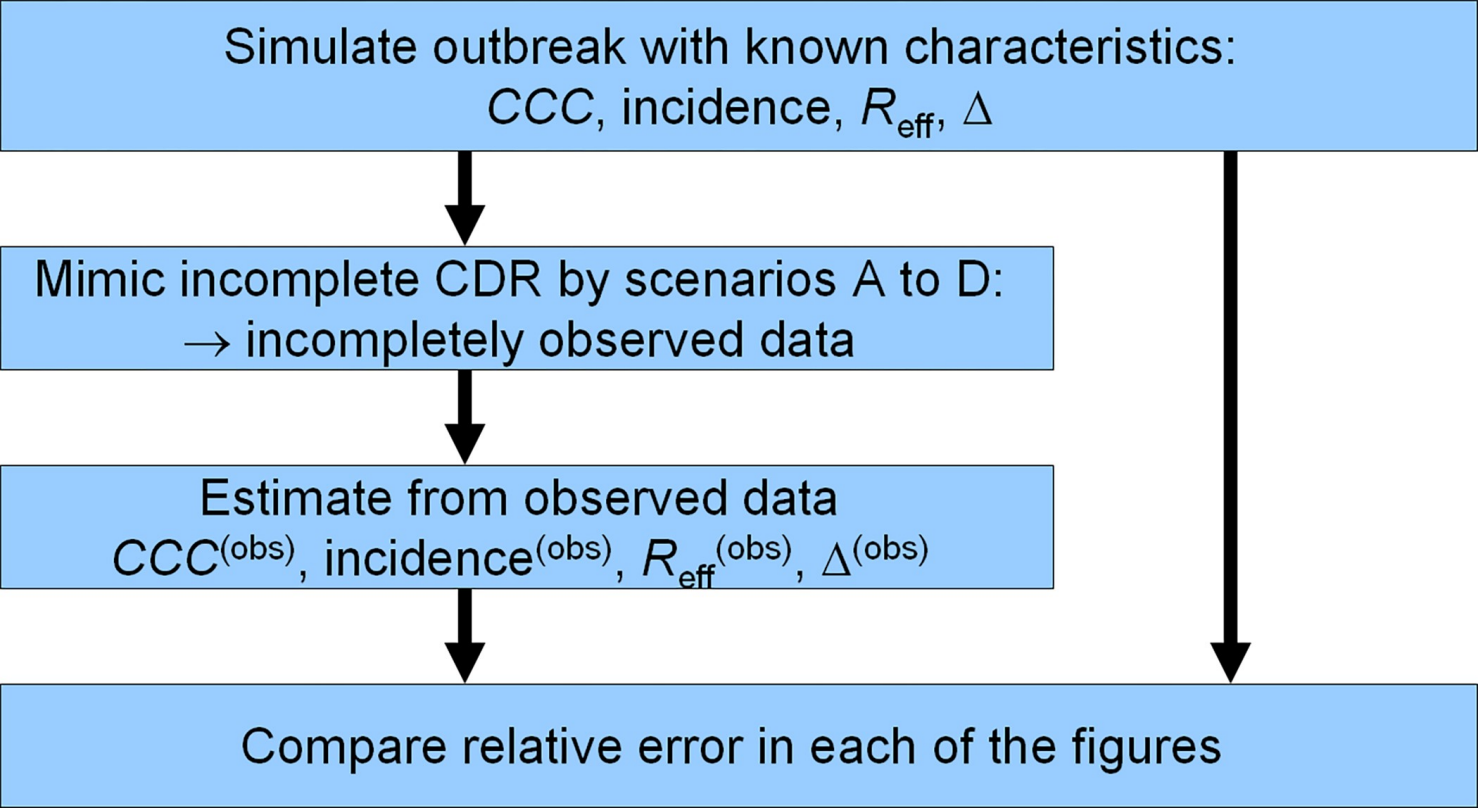
Work flow of the simulation. Work flow for running the simulation and assessing the bias from incomplete case-detection.

After running the simulation, we mimic incomplete case-detection by applying the scenarios A to D about the CDR, which will lead to incomplete case-detection. Then, the four epidemiological measures are estimated in the presence of incomplete case-detection. These estimates are compared to the true values underlying the simulation during the simulated time period of 60 days. Bias is expressed in terms of relative errors, i.e., 100% (T − E)/T, where T and E denote the true value from the simulation and the estimated value from the observed data in the presence of incomplete case-detection, respectively. The numerical values of the relative errors are assessed at days 15, 30, 45 and 60. In summary, the workflow for running the simulation and making the comparisons is shown in Fig 4.

## Results

**Fig 5:**
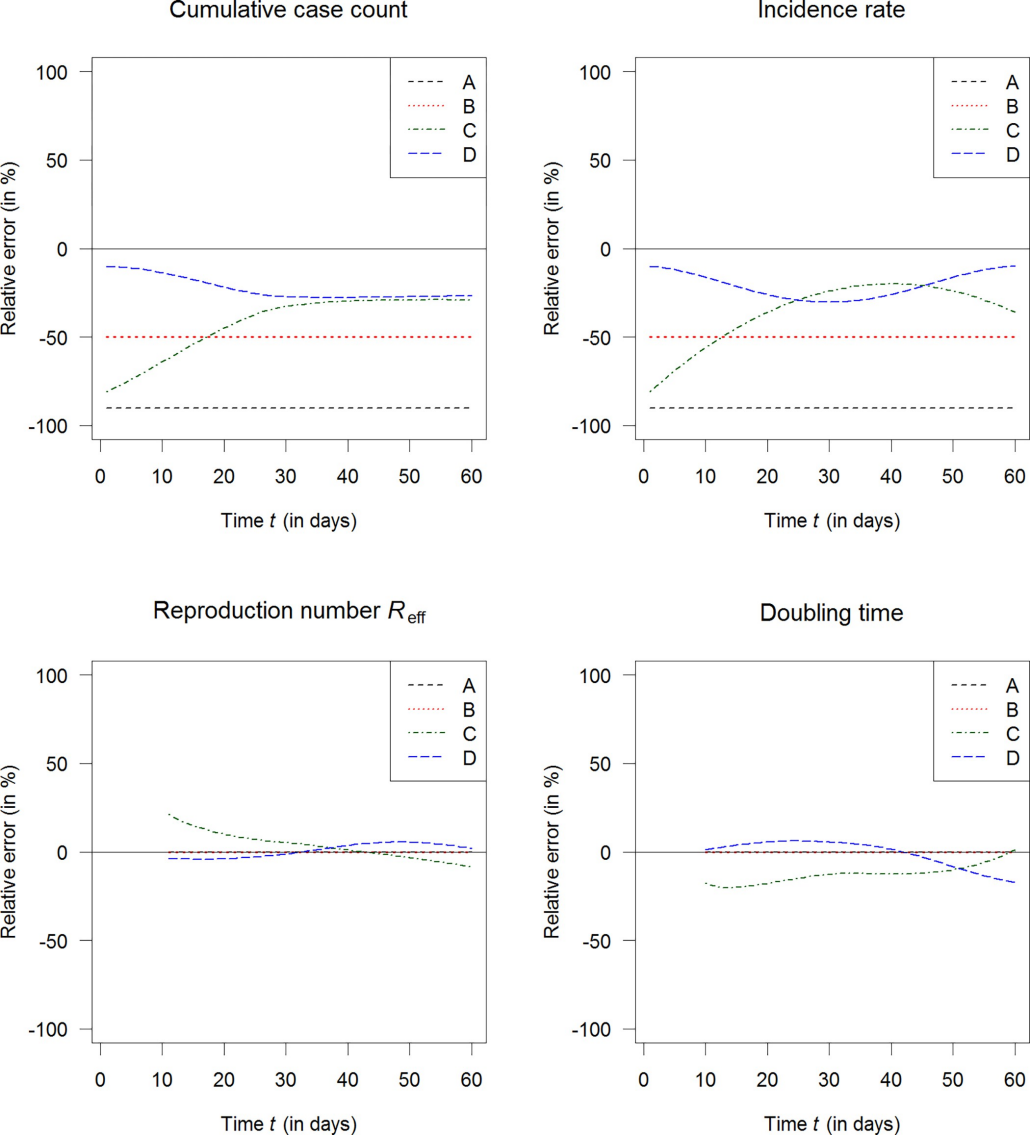
Biases in the simulated scenarios. Bias (relative error) of the four epidemiological measures in the four simulated scenarios A to D (indicated by the line type): Cumulative case count (top left), incidence rate (top right), effective reproduction number (bottom left) and doubling time (bottom right). A desirable zero bias is indicated by a horizontal solid line at value 0.

Fig 5 shows the biases over time in terms of relative errors of the four epidemiological indices from day 0 to day 60 (end of the simulation). A small distance to the horizontal line at value 0 (solid line in each of the four panels) is advantageous. The farther away from the horizontal line at value 0, the higher is the relative error in absolute terms. We see that R_eff_ and the doubling times outperform the CCC and the incidence rate.

Table 1 shows the associated biases in terms of the relative errors at days 15, 30, 45 and 60 of the pandemic. In the table, we observe that in case of the CCC and the incidence rate, none of the relative errors are below 10% in magnitude. In fact, relative errors of CCC and incidence rate can reach up to 90% in magnitude. In all examined scenarios, the effective reproduction number and the doubling time outperform the CCC and the incidence rate. In absolute values, the effective reproduction number is the most unbiased measure while the most frequently used CCC is the least accurate measure in the tested settings.

**Table 1:** Relative errors in the CDR scenarios.

		Relative error (in %)			
Measure	Scenario	Day 15	Day 30	Day 45	Day 60
CCC^a^	A	-90	-90	-90	-90
	B	-50	-50	-50	-50
	C	-54	-33	-29	-29
	D	-18	-27	-27	-27
Incidence	A	-90	-90	-90	-90
	B	-50	-50	-50	-50
	C	-45	-24	-21	-36
	D	-10	-30	-21	-10
R_eff_	A	0	0	0	0
	B	0	0	0	0
	C	15	5	-1	-9
	D	-4	-1	5	2
Doubling time	A	0	0	0	0
	B	0	0	0	0
	C	-20	-13	-12	1
	D	4	6	-3	-17

Relative error (in %) of four epidemiological measures during the hypothetical pandemic in the four scenarios about case detection A to D

^a^cumulative case count

## Discussion

In this work we compared four epidemiological measures that have been used to inform the general public about the SARS-CoV-2 outbreak. Partly, the epidemiological measures entered the public discussion and have been used as a basis for political decisions. For example, national laws in Germany permit school-openings after lockdown if incidence rates are below a certain threshold [1]. Changes of the epidemiological measures have been used to justify interventions such as face masking, travel restrictions and lockdown. Hence, we would expect that the measures are robust with respect to the problem of incomplete case-detection. We found that particularly two measures have not proven to be robust: the frequently used cumulative case counts (CCC) and the incidence rates. The effective reproduction number *R*_eff_ and the doubling times are less prone to imperfect case detection. Although all four measures are interrelated by using the (daily) number of reported cases *F*_t_^(o)^. CCC and incidence rate use *F*_t_^(o)^ in an *absolute* way, either by summing up (CCC) or by relating them to the population (incidence rate). The effective reproduction number *R*_eff_ and the doubling time use *F*_t_^(o)^ in a *relative* way. Fraser’s method to estimate *R*_eff_ is based on a quotient (Equation (11) in S1 File), where the case detection ratio (CDR) cancels out in the numerator and denominator. The doubling time is estimated by the slope of the graph of log(CCC) over time (see Section 3.4 in the S1 File). Estimating the slope in a logarithmic curve is robust with respect to multiplicative changes (because taking the logarithm of a product shifts the curve up- or downwards but does not change the slope). Thus, *R*_eff_ and doubling time are less prone to effects of imperfect case detection.

With a view to these limitations, for future information of the general public, policy makers and national legislators, we recommend to report and place an emphasis on the relative measures effective reproduction number and doubling time in combination with other reported measures such as e.g., hospitalization or intensive care admittance rates. Although we recommend the use of the effective reproduction number and the doubling time, we acknowledge that in the current implementation these measures are estimable no earlier than day 10 after the outbreak, which we think is a minor drawback compared to the possibly high amount of bias by incomplete case-detection in the remaining measures.

The reason for using the infection-age-structured SIR model in our simulation is that this model comprises the SEIR and SIR models as special cases.

Our simulation has a time horizon of 60 days, which is shorter than any real wave of the SARS-CoV-2 pandemic and possibly shorter than a real lockdown. The rationale of this choice was to demonstrate a realistic dynamic range of a wave of the pandemic. Of course, the wave pattern with a phase of a large number of transmissions followed by a phase of lower number of transmissions arises more than once. However, the conclusions of these simulations requires only a realistic range of transmissions, high and low disease activity, no matter what the reasons for these phases with different disease dynamics are (seasonal variations, mask mandates, stay-at-home orders etc). Modeling of different waves, different variants of concern, inclusion of vaccination strategies, possible re-infections, migration, and different fatality rates in different age groups are beyond the scope of this work. We note that in later waves of the pandemic the percentage of asymptomatic cases are likely to be higher than in earlier waves. In terms of the case detection, this means that later waves have a lower CDR than earlier ones, which strikes the importance of reporting the effective reproduction number in successive COVID-19 waves.

Since we are mainly interested in epidemiological measures that assess disease dynamics, we have chosen a relatively simple model without including known risk factors for severe COVID-related complications, such as e.g., age, prevalent co-morbidities or low socio-economic position. Hence, our work should not be interpreted for evaluation of specific interventions or in any prognostic setting. The main focus of the paper are gradual changes in CDRs due to changes in awareness, changes in test policies, availability of test kits etc. Usually, these changes take effect over several days, especially in larger populations. Fluctuations on a daily basis are beyond the scope of our analyses and in extreme cases may lead to very unreliable estimates in any of the four considered epidemiological measures.

Furthermore, we do not discuss practical problems arising with reporting incident cases such as reporting delays, missing data about symptom onset [15] or that lower rates of case-detection are more likely in asymptomatic cases of COVID-19 than in severe cases. However, all four compared epidemiological measures are affected by these problems and our comparison is fair in that respect.

## Conclusions

With a view to future reporting about this or other epidemics, we recommend to use of the effective reproduction number for informing the general public and policy makers. In informing the general public about SARS-CoV-2, we frequently find comparisons between countries. For example, the Corona dashboard of the John Hopkins University simultaneously presents the cumulative case-count for many different counties on a world map [2]. In case that national test strategies differ from one country to another, the possibly biased cumulative case-count or incidence rates leads to erroneous conclusions in cross-country comparisons.

## Supporting information

S1 File Mathematical background. Formulas and description of the infection-age-structured model and the methods of calculating the four epidemiological figures.
(PDF)

S2 File Additional sensitivity analyses. Effects of changes in the temporal pattern of the pandemic and population size.
(DOC)

## List of abbreviations

CCC: cumulative case count
CDR: case-detection ratio
R_eff_: Effective reproduction number
RNA: ribonucleic acid
RT-PCR: reverse transcriptase polymerase chain reaction
SIR model: state model comprising the states susceptible, infected, recovered (SIR)
