# Supplementary material for: Epidemiological measures for assessing the dynamics of the SARS-CoV-2-outbreak: Simulation study about bias by incomplete case-detection
Source: PLoS One. 2022 Oct 26;17(10):e0276311. doi: 10.1371/journal.pone.0276311 (PMC9604981; doi:10.1371/journal.pone.0276311)
Supplement: S1 File — Formulas and description of the infection-age-structured model and the methods of calculating the four epidemiological figures. (PDF) [file pone.0276311.s001.pdf]

Epidemiology and Infection: Epidemiological measures for assessing the dynamics of the SARS-CoV-2-outbreak: simulation study about bias by incomplete case-detection

Brinks R, Küchenhoff H, Timm J, Kurth T, Hoyer A

1 Infection-age structured SIR model

As for SARS-CoV-2 there is evidence that probability of making an effective contact between an infector and a susceptible subject depends on the infector's time since infection [He20], we use the infection-age structured SIR model. Migration, fertility and mortality of non-diseased people plays a minor role in the simulated period of 60 days. Thus, demography of the background host is ignored. Similar to the conventional SIR model (without infection age), the population is partitioned into three states, the *susceptible* state, the *infected* and the *removed* state. The initial letters of the three states give the model's name *SIR*. The *removed* state comprises people recovered and deceased from the *infected* state. The numbers of the people in the susceptible and the removed states at time t are denoted by $S(t)$ and $R(t)$, respectively. Furthermore, let $i(t, \tau)$ denote the density of infected people at time t and duration τ since infection (i.e., the *infection age*), such that the number $I(t)$ of infected at t is

$$I(t) = \int_0^\infty i(t, \tau) \mathrm{d}\tau. \tag{1}$$

The transmission rate of the infected with infection age τ at time t is $\beta(t, \tau)$ and the removal rate from the infectious stage is $\gamma(\tau)$. The rate γ comprises mortality as well as remission.

The SIR model and the rates β and γ controlling the transitions between the states is shown in Figure (1).

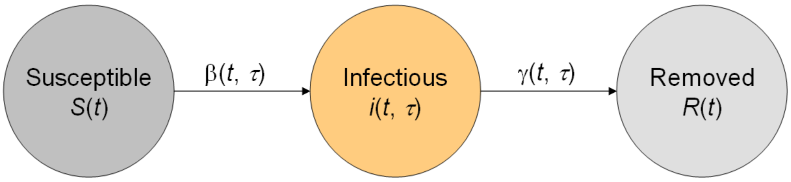

Figure 1: SIR model. The transition rates β and γ depend on the calendar time t and the infection-age τ. The number of people in the respective states *Susceptible, Infectious* and *Removed* are given by S, i, and R.

We can formulate the following model equations for the infection-age SIR model

[Ina17]:

$$\frac{\mathrm{d}S(t)}{\mathrm{d}t} = -\lambda(t)\,S(t) \tag{2}$$

$$\left(\frac{\partial}{\partial t} + \frac{\partial}{\partial \tau}\right) i(t,\tau) = -\gamma(\tau)\,i(t,\tau) \tag{3}$$

$$\frac{\mathrm{d}R(t)}{\mathrm{d}t} = \int_0^\infty \gamma(\tau)\,i(t,\tau). \tag{4}$$

The incidence rate λ in Eq. (2) is given by

$$\lambda(t) = \int_0^\infty \beta(t,\tau)\,i(t,\tau)\mathrm{d}\tau$$

and is usually called *force of infection* [Ina17].

System (2) – (4) is accompanied with initial conditions

$$S(0) = S_0 \tag{5}$$

$$i(t,0) = \lambda(t)\,S(t) \tag{6}$$

$$i(0,\tau) = i_0(\tau) \tag{7}$$

$$i(0,0) = S_0 \int_0^\infty \beta(0,\tau)\,i_0(\tau)\mathrm{d}\tau \tag{8}$$

with positive S_0 and integrable i_0. For later use, we additionally assume that $i(t,\infty) := \lim_{\tau\to\infty} i(t,\tau) = 0$. Condition (8) is called *coupling equation* and guarantees that system (2) – (4) is well-defined [Che16].

Detailed discussion of Equations (2) – (4) with initial conditions (5) – (8) can be found in [Ina17, Chapter 5.3]. In [Ina17, Chapter 5.5] we also find that the age-structured SIR model is a generalization of the frequently used SEIR model.

Using the definition

$$\mathit{\Gamma}(\tau) := \exp\left(-\int_0^\tau \gamma(\sigma)\mathrm{d}\sigma\right), \tag{9}$$

the effective reproduction number $\mathcal{R}_{\mathrm{eff}}(t)$ is given by

$$\mathcal{R}_{\mathrm{eff}}(t) = S(t) \int_0^\infty \beta(t,\tau)\,\mathit{\Gamma}(\tau)\mathrm{d}\tau, \tag{10}$$

[Nis09, Eq. (22),(23)].

2 On the numerical solution of the infection-age SIR model

Assumed $i(t,\tau)$ has to be calculated on a rectangular grid $(t_m,\tau_n) = (m \times \delta_h, n \times \delta_h)$, $m = 0,\ldots,M$, $n = 0,\ldots,N$, as depicted in Figure 2. The grid points are assumed to be equidistant in t- and τ-direction with distance $\delta_h > 0$. A practical strategy for solving Equations (2) – (4) with initial conditions (5) – (8) is given by the following algorithm:

1. Calculate $i(t_m,\tau_n) = i_0(\tau_n - t_m)\,\Gamma(\tau_n)$ for all $n \geq m$. These are the incidence densities at the grid points located on and above the diagonal of the grid (on and above the dashed line in Figure 2).

2. Given that $i(t_m,\tau_n)$ have been calculated on and above the diagonal, set $\ell := 0$ and calculate $\lambda(t_{\ell+1})$ and $S(t_{\ell+1})$ to determine $i(t_{\ell+1},0)$.

3. Calculate $i(t_{\ell+1+k},\tau_k)$, $k = 1,2,\ldots$. The grid points $(t_{\ell+1+k},\tau_k)$ are the points on a subdiagonal. We have $i(t_{\ell+1+k},\tau_k) = i(t_{\ell+1},0)\,\Gamma(\tau_k)$.

4. Set $\ell := \ell + 1$ and repeat steps 2 to 4 until the incidence density i has been calculated on all points (t_m,τ_n) $m = 0,\ldots,M, n = 0,\ldots,N$, on the grid.

Figures 3 and 4 show the resulting incidence density $i(t,\tau)$ obtained from the algorithm presented above and the number $I(t)$ of infective people calculated by (1), respectively. The number of infected people increases steeply until about day $t = 30$ when the lockdown is effective.

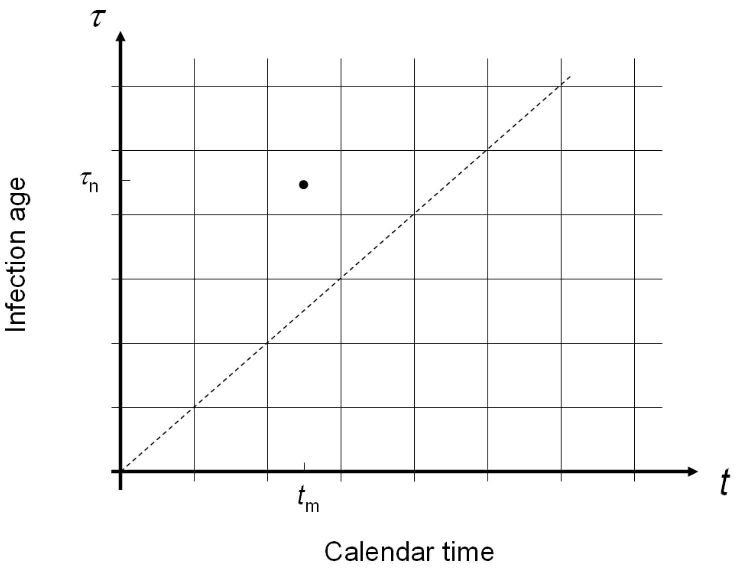

Figure 2: Rectangular grid representing calendar time t (abscissa) and infection-age τ (ordinate). The grid point (t_m, τ_n) above the main diagonal (dashed line) is highlighted.

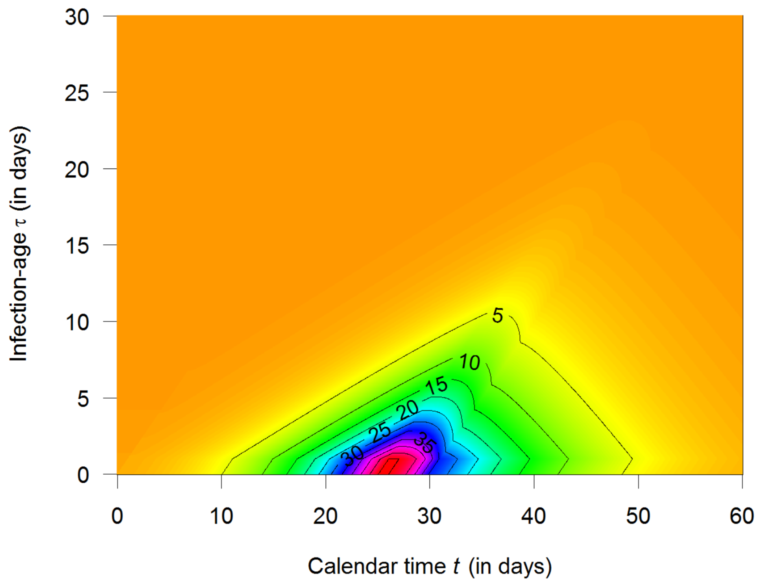

Figure 3: Contour plot of the incidence-density $i(t, \tau)$ (in units 1000).

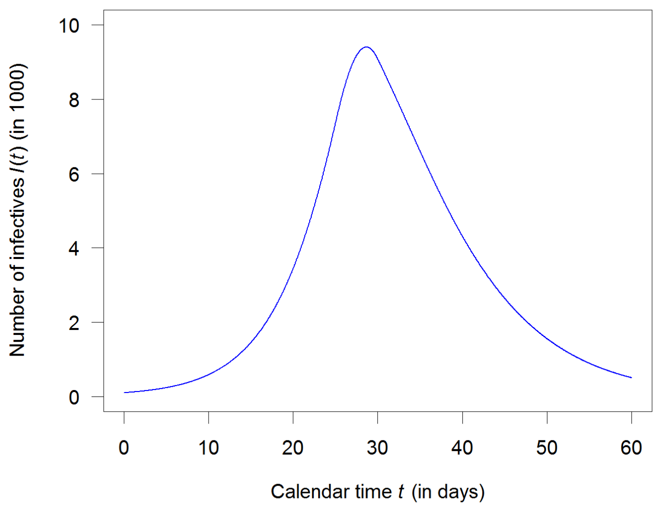

Figure 4: Number I of infected people over the simulated time period (in 1000).

3 Details of calculating the four epidemiologic measures

3.1 Cumulative case counts

The cumulative case count $CCC(t)$ up to time t is the sum of incident cases until day t:

$$CCC(t) = \sum_{s=1}^{t} F_s.$$

Accordingly, the observed cumulative case count, $CCC^{(o)}(t)$ is defined as

$$CCC^{(o)}(t) = \sum_{s=1}^{t} F_s^{(o)}.$$

3.2 Incidence rate

As usual, the incidence rate is defined as the number of incident cases F_t over the population at risk. The size of population at risk is the number of susceptibles S_t. Compared to the number $S(t)$ defined via Equations (2) – (4) an initial conditions (5) – (8), S_t is an daily average of $S(t)$. Similarly, the observed incidence rate is defined via $F_t^{(o)}$ over the associated population at risk. The size of this population at risk is $S_t^{(o)}$, which may not be exactly the same as S_t. For large populations (i.e., large $S + I + R$), we can assume that difference is negligible and $S_t \approx S_t^{(o)}$. Thus, for calculating the relative error E of the incidence rate, we chose to calculate and present $E = \frac{F_t^{(o)} - F_t}{F_t}$.

3.3 Effective reproduction number $\mathcal{R}_{\text{eff}}$

We use the Fraser-method to estimate the instantaneous reproduction number $\mathcal{R}_{\text{eff}}$. The underlying estimation equation is

$$\mathcal{R}_{\text{eff}}(t) = \frac{F_t}{\sum_{j=0}^{J} w_j F_{t-j}}, \tag{11}$$

where w_j are weights with $0 \leq w_j \leq 1, \ j = 0, ..., J$ based on the generation time distribution [Fra07, Eq. (9)]. For estimation of these weights we have chosen $J = 9$ and $w_j = 0$, for $j = 0, 1, 2, \ w_3 = 6/64, w_j = 10/64$, for $j = 4, \dots, 8$, and $w_9 = 8/64$ by discretizing the distribution.

Obviously, the estimation method for $\mathcal{R}_{\text{eff}}(t)$ uses data from the $J = 9$ preceding days $t-9, t-8, \ldots, t$, the earliest day with an available estimate for $\mathcal{R}_{\text{eff}}(t)$ is day $t = 10$. The corresponding estimates of $\mathcal{R}_{\text{eff}}(t)$ based on the daily numbers F_t are shown as solid black line in Figure 5. For comparison, the time-continuous values $\mathcal{R}_{\text{eff}}(t)$ calculated by numerically integrating Eq. (10) are drawn as dashed blue line.

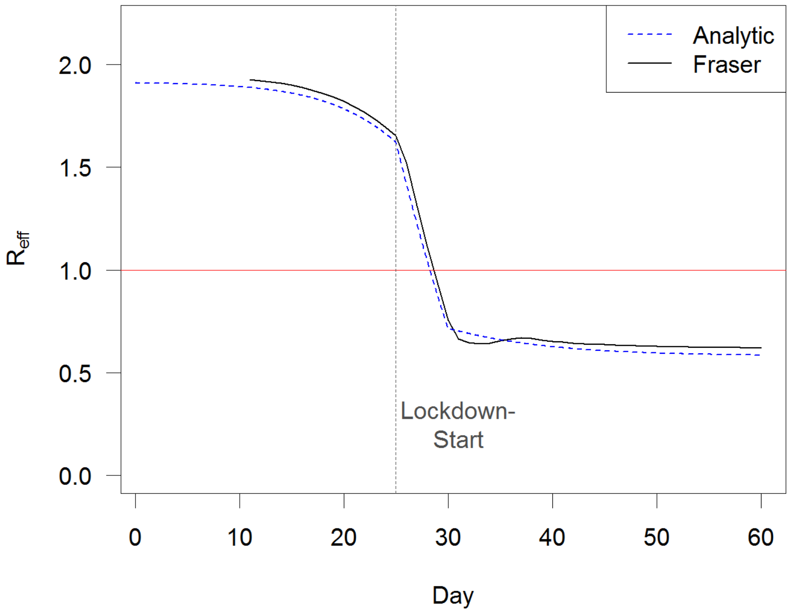

Figure 5: Effective reproduction number $\mathcal{R}_{\text{eff}}(t)$ over time as calculated by Eq. (10) (dashed blue line) and discretized estimates based on the daily numbers F_t using Fraser's method as in Eq. (11) (solid black). The start of the lockdown at day $t = 25$ is indicated by a vertical dashed line (grey).

3.4 Doubling times

As described in the main text, the doubling time Δ at time t is defined by

$$2 \times CCC(t) = CCC\big(t + \Delta(t)\big). \tag{12}$$

For estimating $\Delta(t)$, the cumulative case count $CCC(t)$ at day t is modelled by fitting a linear regression line to the logarithmized case counts of the $J = 9$ previous days, i.e., to the points

$$\Big(t - j, \log\big(CCC(t - j)\big)\Big),\ j = 0, \dots, J.$$

Assumed the associated regression line at day t reads as $a_t + b_t \times t$, then an easy calculation shows that the doubling time $\Delta(t)$ is given by $\Delta(t) = \frac{\log(2)}{b_t}$.

As the estimation method for $\Delta(t)$ uses data from the $J = 9$ preceding days, the earliest day with an estimate is $t = 10$.

4 Choice of the parameters for mimicking the SARS-CoV-2 pandemic

We use the parameters as shown in Table 1 to mimic the spread of the virus in the hypothetical population. The transmission rate β is assumed to be a product of two factors β_τ and β_t. Figure 1 of the main text shows the factors β_t and β_τ.

Table 1: Parameters used in the simulation

Parameter	Meaning	Remark	Source
β	Transmission rate	assumed to be a product of two functions $\beta(t, \tau) = \beta_t(t) \times \beta_\tau(\tau)$	
β_t	factor of β depending on t	mimics a lockdown, chosen such that $\mathcal{R}_{\text{eff}}$ drops from > 1 to < 1 (cf. left part of Figure 2 in the main text)	[Lav20]
β_τ	factor of β depending on τ	follows a Gamma distribution with shape 2 and rate .25 $\Rightarrow$ modal value of 4 and mean 8 (see right part of Figure 2 in the main text)	[He20]
γ	Removal rate	Asymptotics similar to β_τ	[He20]

5 Source code

Source code for running the simulation in the free statistical software R (including solving the age-structured SIR model) can be found in the open public repository Zenodo under DOI `10.5281/zenodo.4750942` [Bri21].

References

[Bri21] Brinks R. Source code to Brinks et al (2021): Epidemiological measures during the SARS-CoV-2-outbreak, Zenodo Repository, DOI `10.5281/zenodo.4750942`

[Che16] Chen Y, Zou S, Yang J (2016) Global Analysis of an SIR Epidemic Model with Infection Age and Saturated Incidence, Nonlin Ana: Real World App 30: 16–31.

[Fra07] Fraser C (2007) Estimating Individual and Household Reproduction Numbers in an Emerging Epidemic. PLoS ONE 2(8): e758.

[He20] He X, Lau EHY, Wu P et al. (2020) Temporal dynamics in viral shedding and transmissibility of COVID-19. Nature Medicine 26, 672-75.

[Ina17] Inaba H (2017) Age-structured Population Dynamics in Demography and Epidemiology, Springer, Singapore.

[Lav20] Lavezzo E, Franchin E, Ciavarella C et al. (2020) Suppression of a SARS-CoV-2 outbreak in the Italian municipality of Vo. Nature DOI 10.1038/s41586-020-2488-1

[Nis09] Nishiura H, Chowell G (2009) The Effective Reproduction Number as a Prelude to Statistical Estimation of Time-Dependent Epidemic Trends, in: Chowell G, Hyman JM, Bettencourt LMA, Castillo-Chavez C: Mathmatical and Statistical Estimation Approaches in Epidemiology, Springer, Dordrecht.
