# Supplementary material for: Epidemiological measures for assessing the dynamics of the SARS-CoV-2-outbreak: Simulation study about bias by incomplete case-detection
Source: PLoS One. 2022 Oct 26;17(10):e0276311. doi: 10.1371/journal.pone.0276311 (PMC9604981; doi:10.1371/journal.pone.0276311)
Supplement: S2 File — Effects of changes in the temporal pattern of the pandemic and population size. (DOC) [file pone.0276311.s002.doc]

Epidemiological measures for assessing the dynamics of the SARS-CoV-2-outbreak: simulation study about bias by incomplete case-detection

Ralph Brinks, Helmut Küchenhoff, Jörg Timm, Tobias Kurth, Annika Hoyer

# Supplementary Material: Sensitivity analyses

In these sensitivity analyses we change the assumptions about 1) the temporal pattern of the simulated pandemic and 2) the initial population size.

## Part 1: Changes in the temporal pattern of the pandemic

In the simulation described in the main text, the phase of increasing number of infections (from *t* = 0 to *t* = 25, in days) is followed by a phase of implementing a lockdown. This wash-in phase lasts from *t* = 25 to *t* = 30. The rationale for this wash-in period with duration W = 5 (days) is that in real populations, public health interventions usually require some time before taking full effect. After *t* = 30 it has been assumed in the main text scenario that the (full) effect remains unaltered until the end of the simulation at *t* = 60. We call this scenario the base-case.

In the sensitivity analyses (SAs), the duration W of the wash-in period has been shorted to W = 2 (SA 1) and prolonged to W = 10 days (SA 2), respectively. In both scenarios, SA 1 and SA 2, it is still assumed that the effect of the lockdown remains unchanged until the end of the simulation at *t* = 60. In the third scenario of the SAs, we assume W = 5 as in the base-case and then a re-emergence of the pandemic after 10 days (at *t* = 40).

| **SA scenario** | **Duration W of wash-in period (in days)** | **Assumed effect of lock-down** |
| --- | --- | --- |
| Base-case | 5 | Control |
| SA 1 | 2 | Control |
| SA 2 | 10 | Control |
| SA 3 | 5 | Re-emergence after 10 days |

**Table S1: Overview of scenarios for the sensitivity scenarios (SAs). The base-case refers to the situation described in the main text.**

The results of the SA scenarios in terms of the relative errors are shown in Table S2. We see that most relative errors remain the same up to 2 percentage points. The few exceptions from ±2 percentage points are seen in SA3 where the effective reproduction number and most of the doubling times still outperforms the other measures in terms of relative errors. Thus, the conclusions drawn in the main text remain valid in the SAs 1 to 3.

|  |  | **CDR Scenario** | **Relative error (in %)** | | | |
| --- | --- | --- | --- | --- | --- | --- |
| **SA scenario** | **Measure** |  | **Day 15** | **Day 30** | **Day 45** | **Day 60** |
| SA 1 | CCC | A | -90 | -90 | -90 | -90 |
|  |  | B | -50 | -50 | -50 | -50 |
|  |  | C | -54 | -34 | -30 | -30 |
|  |  | D | -18 | -27 | -27 | -26 |
|  | Incidence | A | -90 | -90 | -90 | -90 |
|  |  | B | -50 | -50 | -50 | -50 |
|  |  | C | -45 | -24 | -21 | -36 |
|  |  | D | -21 | -30 | -21 | -10 |
|  | R_eff_ | A | 0 | 0 | 0 | 0 |
|  |  | B | 0 | 0 | 0 | 0 |
|  |  | C | 15 | 6 | -1 | -9 |
|  |  | D | -4 | -1 | 5 | 2 |
|  | Doubling  time | A | 0 | 0 | 0 | 0 |
|  |  | B | 0 | 0 | 0 | 0 |
|  |  | C | -20 | -13 | -13 | 1 |
|  |  | D | 4 | 6 | -3 | -17 |
| SA 2 | CCC | A | -90 | -90 | -90 | -90 |
|  |  | B | -50 | -50 | -50 | -50 |
|  |  | C | -54 | -32 | -28 | -28 |
|  |  | D | -18 | -27 | -28 | -27 |
|  | Incidence | A | -90 | -90 | -90 | -90 |
|  |  | B | -50 | -50 | -50 | -50 |
|  |  | C | -45 | -24 | -21 | -36 |
|  |  | D | -21 | -30 | -21 | -10 |
|  | R_eff_ | A | 0 | 0 | 0 | 0 |
|  |  | B | 0 | 0 | 0 | 0 |
|  |  | C | 15 | 5 | -1 | -9 |
|  |  | D | -4 | -1 | 5 | 2 |
|  | Doubling  time | A | 0 | 0 | 0 | 0 |
|  |  | B | 0 | 0 | 0 | 0 |
|  |  | C | -20 | -12 | -11 | 2 |
|  |  | D | 4 | 6 | -3 | -17 |
| SA 3 | CCC | A | -90 | -90 | -90 | -90 |
|  |  | B | -50 | -50 | -50 | -50 |
|  |  | C | -54 | -33 | -29 | -36 |
|  |  | D | -18 | -27 | -27 | -21 |
|  | Incidence | A | -90 | -90 | -90 | -90 |
|  |  | B | -50 | -50 | -50 | -50 |
|  |  | C | -45 | -24 | -21 | -36 |
|  |  | D | -21 | -30 | -21 | -10 |
|  | R_eff_ | A | 0 | 0 | 0 | 0 |
|  |  | B | 0 | 0 | 0 | 0 |
|  |  | C | 15 | 5 | -1 | -8 |
|  |  | D | -4 | -1 | 4 | 2 |
|  | Doubling  time | A | 0 | 0 | 0 | 0 |
|  |  | B | 0 | 0 | 0 | 0 |
|  |  | C | -20 | -13 | -12 | 4 |
|  |  | D | 4 | 6 | -4 | -13 |

Table S2: Results in the different SA scenarios.

## Part 2: Changes in the population size

We changed the population sizes *S*_0_ (cf. Eq. (5) of the technical supplement) of the simulation from the size of small town (*S*_0_ = 10,000) to a bigger country (*S*_0_ = 500 million). Table S3 shows the results of the relative error at day *t* = 60.

| Measure | CDR  scenario | **Relative error (in %) at day 60** | | | | | | | | | |
| --- | --- | --- | --- | --- | --- | --- | --- | --- | --- | --- | --- |
|  |  | S_0_ = 10,000 | S_0_ = 50,000 | S_0_ = 100,000 | S_0_ = 500,000 | S_0_ = 1 mio | S_0_ = 5 mio | S_0_ = 10 mio | S_0_ = 50 mio | S_0_ = 100 mio | S_0_ = 500 mio |
| CCC | A | -90 | -90 | -90 | -90 | -90 | -90 | -90 | -90 | -90 | -90 |
|  | B | -50 | -50 | -50 | -50 | -50 | -50 | -50 | -50 | -50 | -50 |
|  | C | -40 | -31 | -29 | -26 | -26 | -26 | -26 | -26 | -26 | -26 |
|  | D | -24 | -27 | -27 | -25 | -24 | -23 | -23 | -23 | -23 | -23 |
| In- cidence | A | -90 | -90 | -90 | -90 | -90 | -90 | -90 | -90 | -90 | -90 |
|  | B | -50 | -50 | -50 | -50 | -50 | -50 | -50 | -50 | -50 | -50 |
|  | C | -36 | -36 | -36 | -36 | -36 | -36 | -36 | -36 | -36 | -36 |
|  | D | -10 | -10 | -10 | -10 | -10 | -10 | -10 | -10 | -10 | -10 |
| R_eff_ | A | 0 | 0 | 0 | 0 | 0 | 0 | 0 | 0 | 0 | 0 |
|  | B | 0 | 0 | 0 | 0 | 0 | 0 | 0 | 0 | 0 | 0 |
|  | C | -9 | -9 | -9 | -8 | -8 | -8 | -8 | -8 | -8 | -8 |
|  | D | 2 | 2 | 2 | 2 | 2 | 2 | 2 | 2 | 2 | 2 |
| Doubling time | A | 0 | 0 | 0 | 0 | 0 | 0 | 0 | 0 | 0 | 0 |
|  | B | 0 | 0 | 0 | 0 | 0 | 0 | 0 | 0 | 0 | 0 |
|  | C | -15 | -3 | 1 | 6 | 6 | 7 | 7 | 7 | 7 | 7 |
|  | D | -13 | -17 | -17 | -16 | -15 | -15 | -15 | -15 | -15 | -15 |

We can conclude that the results described in the main text are robust with respect to variations in population size.
